# Prevalence and Predictors of Abdominal Aorta Calcification in Patients With Psoriasis—A Case Control Study

**DOI:** 10.3389/fmed.2022.890195

**Published:** 2022-06-30

**Authors:** Sofia Ramos, Sheetal Daya, Nigel J. Crowther, Lushen Pillay, Mohammed Tikly, Nasrin Goolam Mahyoodeen

**Affiliations:** ^1^Department of Diagnostic Radiology, Charlotte Maxeke Johannesburg Academic Hospital, Faculty of Health Sciences, University of the Witwatersrand, Johannesburg, South Africa; ^2^Department of Chemical Pathology, National Health Laboratory Services, Faculty of Health Sciences, University of the Witwatersrand, Johannesburg, South Africa; ^3^Department of Dermatology, Faculty of Health Sciences, Helen Joseph Hospital, University of the Witwatersrand, Johannesburg, South Africa; ^4^Department of Internal Medicine, Faculty of Health Sciences, Chris Hani Baragwanath Academic Hospital, University of the Witwatersrand, Johannesburg, South Africa

**Keywords:** abdominal aortic calcification (AAC), atherosclerosis, smoking, cardiometabolic syndrome (CMetS), psoriasis

## Abstract

**Background:**

Psoriasis is associated with a high prevalence of cardiovascular disease in Caucasians, but only a few studies from sub-Saharan Africa have investigated the prevalence of cardiovascular disease in patients with psoriasis. Abdominal aortic calcification (AAC) is a strong predictor of future cardiovascular events and all-cause mortality in the general population. We investigated the prevalence and risk factors for AAC in a predominantly non-Caucasian cohort of South African patients with psoriasis.

**Methods:**

A cross-sectional case-control study of adult psoriasis patients (*n* = 69) and controls (*n* = 80), matched for gender, ethnicity and body mass index, attending tertiary Dermatology and Rheumatology clinics in Johannesburg, South Africa. Demographic, anthropometric, clinical and biochemical data were recorded. All participants underwent non-contrast abdominal CT scans. Images were assessed for AAC at the supra-coeliac aorta, supra-mesenteric aorta and aortic bifurcation using Horos DICOM viewer software.

**Results:**

Abdominal aortic calcification at any site was more prevalent in the psoriasis than control group (47.8% vs 22.5%, *p* < 0.005). The aortic bifurcation was the commonest site for AAC in both groups, but more prevalent in the psoriasis group (42.0% vs 21.3%, *p* < 0.005). The psoriasis group was also more likely to smoke, have hypertension and type 2 diabetes (56.5% vs 25.0%, *p* < 0.005; 72.0% vs 55.0%, *p* < 0.005; 24.6% vs 3.80%, *p* < 0.0005, respectively). Multivariable logistic regression analysis demonstrated that age, smoking and type 2 (T2DM) diabetes were independently associated with AAC (odds ratio (95% CIs): 1.16 (1.07, 1.20), 4.30 (2.15, 8.61) and 3.45 (1.09, 15.7) respectively), but psoriasis was not. Forward regression analysis demonstrated that smoking attenuated the association of psoriasis with AAC.

**Conclusion:**

Our findings show AAC to be more common in psoriasis patients compared to controls. Age, T2DM and smoking were independent predictors of AAC. Smoking cessation is essential in psoriatic patients to reduce the risk of cardiovascular events. The clinical utility of AAC detection by CT imaging to risk stratify for hard cardiovascular outcomes needs to be explored.

## Background

Psoriasis is a chronic immune-mediated inflammatory skin disorder (1) characterized by raised, erythematous plaques with adherent, silvery scales (2). Several studies, in mainly Caucasian populations, have shown an increased prevalence of metabolic syndrome (MetS) and its components, i.e., hypertension, dyslipidaemia, and type 2 diabetes mellitus (T2DM) in psoriasis. Consequently patients with psoriasis have a higher risk of cardiovascular disease compared to the general population (3), similar to that observed in patients with rheumatoid arthritis and T2DM (4). A recent South African study has shown a higher prevalence of the metabolic syndrome and its components in patients with psoriasis compared with controls (5). The increased risk of cardiovascular disease is mediated not only by a higher prevalence of traditional risk factors such as smoking (6), obesity and insulin resistance in psoriasis (3), but also by the systemic pro-inflammatory milieu of the disease (7).

Atherosclerotic plaques are a strong predictor of cardiovascular events (8) in the general population. Cytokine up-regulation, a hallmark of psoriasis, and production of pro-angiogenic factors, accelerates atherosclerosis and plaque formation (9). The severity and extent of atherosclerotic plaques in psoriasis correlates with disease activity and traditional risk factors (10). To date, ultrasonography is the most widely used imaging modality for atherosclerotic plaque formation. The American College of Cardiology and American Heart Association guidelines now recommend computed tomography (CT) based imaging for the assessment of atherosclerotic cardiovascular disease (11). The use of non-contrast enhanced multidetector CT (MDCT) provides highly sensitive and reliable detection of calcified atherosclerosis (12).

Atherosclerosis in the abdominal aorta occurs most frequently at the distal aorta and bifurcation, resulting from turbulent flow in this region (13). Plaque within this region is also associated with the highest probability of symptomatic atherosclerosis at other sites and shows the highest tendency for recurrence (14). To date, there is a paucity of data on the prevalence of AAC in psoriasis, with no data from sub-Saharan Africa. To further compound this, there is very little data regarding psoriasis and cardiometabolic risk factors in sub-Saharan populations, with most of the available literature regarding psoriasis outcomes coming from developed countries (15). Local data has demonstrated a high burden of cardiometabolic disease (CMD), especially amongst those with severe psoriasis (5, 16). There is no data examining the prevalence of atherosclerosis within this specific subset of patients. With this in mind, the present cross-sectional study sought to determine the prevalence and predictors of AAC in adult South African patients with established plaque psoriasis.

## Methods

As part of a larger study on psoriasis and CMD, adult psoriasis patients (*n* = 69) and controls (*n* = 80) were recruited at tertiary public service Dermatology and Rheumatology clinics in Johannesburg, South Africa. Appropriate ethical clearance was received. Patients were 18 years or older, and had a diagnosis of psoriasis or psoriatic arthritis (PsA), were HIV seronegative and had no other immune-mediated inflammatory diseases (IMIDs). Control participants had no history of any IMIDs, were of a similar socio-geographic background, were matched for gender, ethnicity and body mass index (BMI).

Clinical and laboratory data were obtained by review of case records and a once-off clinical examination and a fasting blood sample. The patient variables included: age, ethnicity (black African, Asian, Caucasian, mixed ancestry), gender, level of education (completing high school or less), smoking status, BMI, blood pressure (mean blood pressure of at least two readings taken 10 minutes apart with the participant seated). Waist circumference was measured at the mid-point of the upper border of the iliac crest and the lower border of the last rib using a soft tape measure.

Laboratory investigations included a fasting glucose and serum lipid profile. Venous blood samples were obtained at the time of clinical examination after an overnight fast. Plasma glucose and serum lipids (total cholesterol, triglycerides and high-density lipoprotein (HDL) cholesterol), were measured using enzymatic methods on the ADVIA 1800 Chemistry Systems Analyser (Siemens Healthcare Diagnostics, Tarrytown, NY, USA). Low-density lipoprotein (LDL) cholesterol was calculated using the Friedewald formula (17).

The MetS was diagnosed according to the 2009 harmonized guidelines (18). In patients without a history of T2DM and hypertension, newly diagnosed T2DM was based on a fasting plasma glucose of >7mmol/l (19) and newly diagnosed hypertension based on the blood pressure criteria within the harmonized guidelines (18). High LDL was defined according to the South African Heart Association and Lipid and Atherosclerosis Society of Southern Africa guidelines (20). More detailed descriptions of the methods used in this study are available in previous publications (5, 21).

Non-contrast abdominal CT imaging was performed at the level of L4/L5, using a Philips 16 slice multidetector CT (Brilliance 16, Philips, Netherlands), with slice thickness of 3 mm (250 mAs and 120kV, FOV 350 mm), within a month of the clinical assessment. Standard resolution (matrix 512 ×512); sharp (C) filter was applied with window range set at 50-350. Subcutaneous and visceral fat volumes, expressed as g/cm^3^, were calculated from CT image data and by using fat selection technology on Osirix Dicom viewer (Osirix foundation, Geneva Switzerland). For the vascular studies, Horos DICOM Viewer version 3.3.0 (GNU L-GPL, Nimble Co LLC d/b/a Purview in Annapolis, MD USA), a Mac-based image processing application, was used to analyse 149 available CT image stacks for presence and location of calcium in the abdominal aorta. The aortic bifurcation was the primary site for evaluation. The principal investigator (SR) read the images twice to assess intra-observer variability and a second radiologist (SD) performed an independent reading to determine inter-observer variability. Other abdominal aortic segments were also visible in several patient image stacks and were therefore also assessed. The secondary sites were the supra-coeliac aorta and supra-mesenteric aorta.

## Statistical Analysis

Student's unpaired t test was used to compare continuous variables between groups, with log transformation of data that was not normally distributed. Categorical variables were compared across groups using the χ^2^ test. Logistic regression analysis was used to determine the independent predictors of aortic calcification. Thus, study variables that correlated with aortic calcification at *p* < 0.20 in univariate logistic regression models were included in a multivariable model. Backward, stepwise removal of non-significant variables from the multivariable model was performed until only those with *p* < 0.05 remained. In the models where psoriasis did not continue through to the final model, forward regression analysis was performed to determine which variable was responsible for attenuating its effect on the outcome variable of calcification. The variables chosen for addition to the forward regression model were any of those included in the initial multivariable model and particularly those that continued through to the final model. All analyses were performed using Statistica version 13.5 (StatSoft, Tulsa, OK, USA).

## Results

Most patients were middle-aged with a mean (SD) age of 53.3 (14.5) years and long-standing disease. Mean (SD) disease duration was 18.9 (13.3) years, with an almost equal gender representation (Table 1). Over 60% of patients were either of Asian or mixed ancestry. The prevalence of smoking, MetS, hypertension and T2DM was significantly higher in the psoriasis group compared to the control group (56.5% vs. 25.0%, *p* < 0.005; 56.5% vs. 37.5%, *p* < 0.05; 72.5% vs. 55.0%, *p* < 0.005; 24.6% vs. 3.80%, *p* < 0.0005, respectively). There were no significant differences in serum lipids or abdominal fat.

**Table 1:** Clinical characteristics of patients and controls.

**Variable**	**Psoriasis group (*n =* 69)**	**Controls group (*n =* 80)**	***p*-value**
Age in years - mean ± SD	53.3 ± 14.5	47.9 ± 14.5	0.02
Ethnicity			
Black	9 (13.0)	15 (18.8)	NS
White	8 (11.6)	12 (15.0)	
Asian	29 (42.0)	32 (40.0)	
Mixed ethnicity	23 (33.3)	21 (26.3)	
Male	32 (46.4)	32 (40.0)	NS
Disease duration in years	18.9 ± 13.3	–	
Smokers	39 (56.5)	20 (25.0)	<0.0001
Metabolic syndrome	39 (56.5)	30 (37.5)	0.02
Hypertension	50 (72.5)	44 (55.0)	0.03
Type 2 diabetes	17 (24.6)	3 (3.80)	0.0002
High LDL-C	52 (75.4)	52 (65.0)	NS
Low HDL-C levels	21 (30.4)	19 (23.8)	NS
Hypertriglyceridaemia	21 (30.4)	18 (22.1)	NS
BMI (kg/m^2^)	32.4 ± 9.38	29.7 ± 6.94	NS
Visceral fat (g/cm^3^)	197.8 (132.5, 277.7)	152.6 (89.5, 218.3)	NS
Subcutaneous fat (g/cm^3^)	439.4 (323.0, 647.8)	410.0 (267.7, 595.9)	NS

*Data given as mean ± SD, median (interquartile range) or n (%); NS, not significant*.

The aortic bifurcation was the commonest site for AAC in both groups (Table 2) and AAC was more prevalent at all sites in the psoriasis group than the control group (aortic bifurcation 48.5% vs. 23.2%, *p* < 0.005; supra-coeliac aorta 21.2% vs. 3.8%, *p* < 0.005; supramesenteric aorta 24.3% vs. 2.3%, *p* < 0.05). Subgroup analysis revealed no difference in AAC between psoriasis patients with or without psoriatic arthritis.

**Table 2:** Abdominal aortic calcification in psoriasis patients vs. controls.

**Variable**	**Psoriasis *n* (%)**	**Controls *n* (%)**	**Odds Ratio (95% CI)**	***p*-value**
Aortic bifurcation	33/68 (48.5)	16/69 (23.2)	3.12 (1.50, 6.51)	0.002
Supra-coeliac area	14/66 (21.2)	3/79 (3.8)	6.82 (1.87, 24.92)	0.003
Supra-mesenteric area	9/37 (24.3)	1/43 (2.3)	13.50 (1.62, 112.54)	0.005

Table 3 is a summary of predictors for AAC at the aortic bifurcation for the entire cohort of patients and controls. Univariate analysis showed a significantly higher burden of traditional cardiovascular risk factors i.e., smoking history, T2DM, hypertension and metabolic syndrome in males and psoriasis patients. Multivariable logistic regression analysis showed age, smoking and T2DM to be independent predictors of AAC at the aortic bifurcation.

**Table 3:** Predictors of abdominal aortic bifurcation calcification.

	**Aortic bifurcation calcification**		**Univariate analysis**		**Multivariate analysis**	
	**Present *n* (%)**	**Absent *n* (%)**	**OR (95% CI)**	***p*-value**	**OR (95% CI)**	***p*-value**
Age in years	61.1 (8.6)	45.5 (13.6)	1.13	<0.0001	1.14	<0.0001
			(1.08, 1.17)		(1.07, 1.20)	
Ethnicity						
Black	3 (6.1)	16 (18.2)	0.29 (0.08, 1.06)	NS	–	–
White	10 (20.4)	9 (10.2)	2.25 (0.85, 5.99)	NS	–	–
Asian	24 (49.0)	35 (39.8)	1.45 (0.72, 2.94)	NS	–	–
Mixed ancestry	12 (24.5)	28 (31.8)	0.70 (0.31,1.53)	NS	–	–
Males	28 (57.1)	31 (35.2)	2.8	0.005	–	–
			(1.36, 5.80)			
Psoriasis	33 (67.3)	35 (39.8)	3.12	0.002	–	–
			(1.50, 6.51)			
Psoriatic arthritis	10 (45.5)	12 (54.5)	1.62	NS	–	–
			(0.64, 4.09)			
Smoking	31 (63.3)	25 (28.4)	4.34	<0.0001	4.3	<0.0001
			(2.06, 9.12)		(2.15, 8.61)	
Metabolic syndrome	32 (65.3)	33 (37.5)	3.18	0.002	–	–
			(1.51, 6.51)			
Hypertension	37 (75.5)	49 (55.7)	2.45	0.02	–	–
			(1.13, 5.33)			
Type 2 diabetes	14 (28.6)	6 (6.8)	6.03	0.0006	4.14	0.035
			(2.16, 16.85)		(1.09, 15.6)	
High LDL-C^*^	44 (91.7)	56 (63.6)	5.89	0.002	–	–
			(1.91, 18.2)			
Low HDL-C	15 (30.6)	22 (25.0)	1.32	NS	–	–
			(0.61, 2.88)			
Hypertriglyceri-	16 (39.0)	18 (20.5)	1.32	NS	–	–
daemia			(0.86, 4.16)			
BMI (kg/m^2^)	32.2 (8.28)	30.1 (7.94)	1.03	NS	–	–
			(0.99, 1.07)			
Visceral fat (g/cm^3^)	256.3	157.6	1.08	<0.0001	–	–
	(163.8, 345.5)	(89.5, 207.2)	(1.04, 1.11)			
						
Subcutaneous fat (g/cm^3^)	493.6	465.7	1.99	NS	–	–
	(279.0, 621.3	(287.3, 600.1)	(0.99, 1.02)			
Level of Education	25 (51.0)	63 (71.6)	0.43	0.02	–	–
			(0.21, 0.90)			

*Data given as mean ± SD, median (interquartile range) or n(%), NS, not significant*.

While psoriasis was significantly associated with aortic bifurcation calcification in the univariate analysis [OR (95% CI): 3.12 (1.50, 6.51); *p* = 0.002], it did not remain significant in the multivariable model [0.992 (0.31, 3.15); *p* = 0.99]. This model was further analyzed using forward regression analysis to determine which independent variable was responsible for attenuating the effect of psoriasis on aortic bifurcation calcification. This showed that smoking was the principal covariate that attenuated the relationship between psoriasis and aortic bifurcation calcification [2.12 (0.93, 4.83); *p* = 0.07 (for psoriasis); 3.31 (1.92, 5.69); *p* < 0.0001 (for smoking)].

## Discussion

In this case-control, multi-ethnic study of AAC in South African participants, as detected by CT imaging, we found the prevalence of AAC to be especially common at the aortic bifurcation in both the psoriasis and control groups. Psoriasis patients had a significantly higher prevalence of AAC at all three aortic anatomical sites, i.e., aortic bifurcation, supracoeliac and supramesenteric. Most of the risk of AAC was associated with traditional cardiometabolic factors, notably smoking, T2DM and age.

Subclinical atherosclerosis in psoriasis has been well-documented in several non-invasive studies. Ultrasound imaging has consistently shown increased carotid intimal thickening and increased risk of carotid plaques in both psoriasis and PsA (22–24). A more recent cross-sectional study of coronary artery calcification (CAC) determined by CT imaging, showed that patients with moderate to severe psoriasis had similar scores to patients with T2DM after adjusting for confounders (25). The increased incidence of atherosclerotic disease is psoriasis, and many autoimmune diseases, is thought to be a result of chronic inflammation which drives endothelial and metabolic dysfunction, and causes lipoprotein aberrations. The psoriatic march concept has increased the focus on molecular and cellular processes responsible for driving inflammation in psoriasis. Psoriasis is now recognized as a T-cell mediated disorder, driven by a pro-inflammatory cascade. The T cell subtypes implicated in the pathogenesis of psoriasis are also found to encourage atherosclerosis (26). Novel research into the anti-aging and anti-inflammatory gene, Sirtuin 1 (SIRT1), has shown decreased levels in patients with MetS, T2DM and psoriasis, further supporting the pro-inflammatory alterations that underly psoriasis (27, 28). SIRT1 downregulation has also been linked to vascular smooth muscle inflammation that contributes to vascular disease processes (29).

Plaques at the distal aorta and bifurcation are associated with the highest probability of symptomatic atherosclerosis at other sites (14). Moreover, numerous studies in the general population from developed countries have shown that AAC is an independent predictor of cardiovascular and all-cause mortality, irrespective of the Framingham risk score (30–34). In the prospective US Multi-Ethnic Study of Atherosclerosis, both CAC and AAC independently predicted coronary heart disease, but AAC was the only independent predictor of cardiovascular mortality and a better predictor of all-cause mortality than CAC (34). From a health economics perspective, AAC in a community-based observational study in men was found to predict overall health costs, independent of prevalent clinical cardiovascular disease (35).

In the present study, psoriasis was associated with AAC only in the univariate analysis. The multivariate analysis subsequently demonstrated that the effects of psoriasis on AAC are, in fact, mediated by smoking. Smoking is known to accelerate atherosclerosis through its induction of oxidative stress, which leads to lipid abnormalities, platelet activation, chronic inflammation and endothelial dysfunction (36, 37). It should be noted that a higher prevalence of smoking has been observed in psoriatic patients (38), and smoking is associated with an increased risk for onset of psoriasis (37). Smoking status is also positively associated with the severity of psoriasis and impacts the potential response to treatment (39, 40). A meta-analysis demonstrated that the odds ratio for psoriasis in smokers is 1.78 (95% CI 1.53–2.06) (37). Data from the Nurses' Health Study have similarly shown a 15–20% risk of psoriasis in smokers (41). The relationship between smoking and psoriasis is complex. Data from Chinese studies suggest an interplay between genes related to smoking habits and those responsible for susceptibility to incident psoriasis (42). The oxidative stress caused by smoking, along with free radical generation, is hypothesized to interfere with signal pathways associated with developing psoriasis; these include mitogen-activated protein kinase, nuclear factor kappa B (NF-κB), and JAK-STAT pathways. Nicotine is known to induce cytokine production, specifically increasing levels of tumor necrosis factor (TNF), interleukin-12 (IL-12), interleukin-2 (IL-2), and granulocyte-monocyte colony-stimulating factor, all of which are considered central to the pathophysiology of psoriasis (41). Furthermore, cigarette smoke has been shown to suppress SIRT1 activity, contributing to chronic inflammation in psoriasis patients who already exhibit negative alterations in SIRT1 expression (27, 43, 44).

Psoriasis is associated with additional cardiometabolic diseases such as MetS, and in particular, T2DM (16, 45–49) in addition to previously mentioned cardiovascular risk factors, such as smoking (2, 37, 41, 50). This has also been demonstrated within our sub-Saharan demographic of psoriasis patients (5, 16). As discussed, studies demonstrate an association between psoriasis and atherosclerosis (46, 51, 52) with one such study demonstrating increased vascular inflammation in psoriasis using PET/CT imaging (53). There is some contradictory evidence concluding that psoriasis is not associated with atherosclerosis, however, this study comprised psoriasis patients with only mild disease (54). To our knowledge, this is the first study to demonstrate that the heightened risk of AAC in South African psoriasis patients, is explained by smoking. This serves as an important finding as other international studies have similarly shown that the higher risk of stroke and myocardial infarction in psoriasis is due to the high prevalence of other risk factors, rather than psoriasis being the causative agent (21, 54–56).

## Limitations and Conclusion

The limitations of the present study were the cross-sectional design and small sample size, which did not allow us to examine the relationship of AAC with hard cardiovascular outcomes and mortality in psoriasis. Non-contrast CT imaging may potentially have underestimated the prevalence and extent of AAC as compared to CT imaging with intravenous contrast. Notwithstanding these limitations, the strength of the study is the use of a sensitive, robust and reliable diagnostic method of detecting AAC. The clinical utility of this imaging modality as a predictor of cardiovascular and all-cause mortality requires a larger prospective study. From a bedside perspective, our findings underscore the need to address modifiable risk factors, in particular smoking, to mitigate both the burden of psoriasis and attendant cardiovascular risks. Smoking further compromises response and adherence to treatment, highlighting the importance of advocating for smoking cessation in this population.

## Data Availability Statement

The original contributions presented in the study are included in the article/supplementary material, further inquiries can be directed to the corresponding author/s.

## Ethics Statement

The studies involving human participants were reviewed and approved by Human Research Ethics Committee, University of the Witwatersrand. The patients/participants provided their written informed consent to participate in this study.

## Author Contributions

SR: conceptualization of study, radiological data interpretation, statistical analysis, and writing of manuscript. SD: conceptualization of study, radiological data interpretation, and review of final manuscript. NC: conceptualization of study, data interpretation, statistical analysis, and writing of manuscript. LP: conceptualization of study and review of final manuscript. NG: conceptualization of study, data acquisition, data interpretation, statistical analysis, and writing of manuscript. MT: conceptualization of study, data interpretation, and writing of manuscript. All authors approved the final manuscript submitted for publication and agree to be accountable for all aspects of the work, ensuring the accuracy, and integrity of the publication.

## Funding

This work was supported by grants from the Carnegie Corporation of New York, NY, USA. Grant Number: B 8749.RO1 to NG, the National Research Foundation (Thuthuka to NG), the Astra Zeneca Research Trust (to NG), and the Medical Research Council of South Africa (Self-initiated Research Grant to MT). The funders had no role in the design of the study or the collection, analysis and interpretation of data, writing or approval of the manuscript, and decision to submit the manuscript for publication.

## Conflict of Interest

The authors declare that the research was conducted in the absence of any commercial or financial relationships that could be construed as a potential conflict of interest.

## Publisher's Note

All claims expressed in this article are solely those of the authors and do not necessarily represent those of their affiliated organizations, or those of the publisher, the editors and the reviewers. Any product that may be evaluated in this article, or claim that may be made by its manufacturer, is not guaranteed or endorsed by the publisher.

## Abbreviations

AAC: Abdominal aortic calcification
BMI: Body mass index
CAC: Coronary artery calcification
CI: Confidence interval
CMD: Cardiometabolic disease
CT: Computed tomography
CVD: Cardiovascular disease
HDL: High-density lipoprotein
HIV: Human immunodeficiency virus
IMID: Immune-mediated inflammatory disease
LDL: Low-density lipoprotein
MetS: Metabolic syndrome
OR: Odds ratio
PASI: Psoriasis area and severity index
PsA: Psoriatic arthritis
SD: Standard deviation
SIRT1: Sirtuin 1
T2DM: Type 2 diabetes mellitus.
